# (20*S*)-22-Acetoxymethyl-6*β*-meth­oxy-3α,5-dihydro-3′*H*-cyclo­propa[3α,5]-5α-pregnane

**DOI:** 10.1107/S1600536809020674

**Published:** 2009-06-06

**Authors:** Kamal Aziz Ketuly, A. Hamid A. Hadi, Seik Weng Ng

**Affiliations:** a Department of Chemistry, University of Malaya, 50603 Kuala Lumpur, Malaysia

## Abstract

In the title steroid derivative, C_25_H_40_O_3_, the fused cyclo­propane unit that corresponds to a part of the *A* ring has a β-configuration and the associated cyclo­pentane ring an envelope-shaped conformation.

## Related literature

For the synthesis and crystal structure of the iodo-substituted compound, see: Ketuly *et al.* (2009 ▶). The absolute configuration of the acetoxymethyl title compound is that of the iodo-substituted compound.
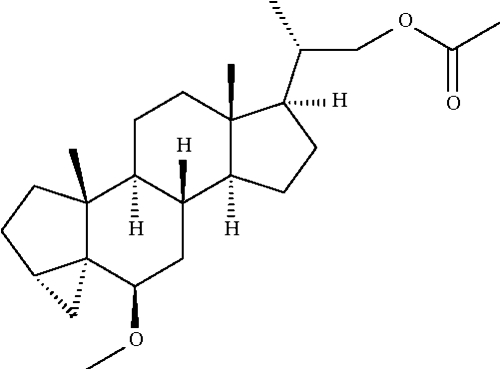


## Experimental

### 

#### Crystal data

C_25_H_40_O_3_
*M* *_r_* = 388.57
Monoclinic, 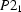

*a* = 9.8222 (1) Å
*b* = 7.6128 (1) Å
*c* = 15.2309 (2) Å
β = 107.604 (1)°
*V* = 1085.55 (2) Å^3^
*Z* = 2
Mo *K*α radiation
μ = 0.08 mm^−1^
*T* = 100 K
0.30 × 0.10 × 0.02 mm

#### Data collection

Bruker SMART APEX diffractometer
Absorption correction: none
10411 measured reflections
2674 independent reflections
2550 reflections with *I* > 2σ(*I*)
*R*_int_ = 0.023

#### Refinement

*R*[*F* ^2^ > 2σ(*F* ^2^)] = 0.033
*wR*(*F* ^2^) = 0.092
*S* = 1.05
2674 reflections
258 parameters
1 restraint
H-atom parameters constrained
Δρ_max_ = 0.30 e Å^−3^
Δρ_min_ = −0.18 e Å^−3^

### 

Data collection: *APEX2* (Bruker, 2008 ▶); cell refinement: *SAINT* (Bruker, 2008 ▶); data reduction: *SAINT*; program(s) used to solve structure: *SHELXS97* (Sheldrick, 2008 ▶); program(s) used to refine structure: *SHELXL97* (Sheldrick, 2008 ▶); molecular graphics: *X-SEED* (Barbour, 2001 ▶); software used to prepare material for publication: *publCIF* (Westrip, 2009 ▶).

## Supplementary Material

Crystal structure: contains datablocks global, I. DOI: 10.1107/S1600536809020674/xu2534sup1.cif

Structure factors: contains datablocks I. DOI: 10.1107/S1600536809020674/xu2534Isup2.hkl

Additional supplementary materials:  crystallographic information; 3D view; checkCIF report

## Figures and Tables

**Fig. 1.**
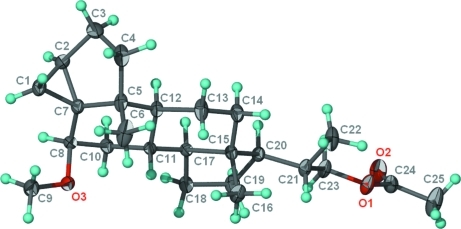
Thermal ellipsoid plot (Barbour, 2001) of C25H40O3 at the 70% probability level. Hydrogen atoms are drawn as spheres of arbitrary radius.

## supplementary crystallographic information

### Experimental

(20*S*)-22-Iodomethyl-6-methoxy-3,5-dihydro-3'*H*-cyclopropa[3α,5]-5-pregnane (Ketuly *et al.*, 2009) (250 mg) was dissolved in pyridine (2 ml) and acetic anhydride (2 ml). The solution was heated at 353 K for an hour. The solvent was evaporated to give a product (280 mg), which was purified by recrystallization from ethanol (140 mg); m.p. 395–396 K. Mass spectrum: M^+^ 338. C&H elemental analysis: calc. C 77.27, H 10.38% (found: C 77.06, H 10.44%).

### Refinement

Hydrogen atoms were placed at calculated positions (C–H 0.95–0.99 Å) and were treated as riding on their parent carbon atoms, with *U*(H) set to 1.2–1.5 times *U*_eq_(*C*). 2271 Friedel pairs were merged.

### Crystal data

**Table:**

C_25_H_40_O_3_	*F*(000) = 428
*M**_r_* = 388.57	*D*_x_ = 1.189 Mg m^−^^3^
Monoclinic, *P*2_1_	Mo *K*α radiation, λ = 0.71073 Å
Hall symbol: P 2yb	Cell parameters from 5999 reflections
*a* = 9.8222 (1) Å	θ = 2.2–28.3°
*b* = 7.6128 (1) Å	µ = 0.08 mm^−^^1^
*c* = 15.2309 (2) Å	*T* = 100 K
β = 107.604 (1)°	Plate, colorless
*V* = 1085.55 (2) Å^3^	0.30 × 0.10 × 0.02 mm
*Z* = 2	

### Data collection

**Table:**

Bruker SMART APEX diffractometer	2550 reflections with *I* > 2σ(*I*)
Radiation source: fine-focus sealed tube	*R*_int_ = 0.023
graphite	θ_max_ = 27.5°, θ_min_ = 2.2°
ω scans	*h* = −12→12
10411 measured reflections	*k* = −9→9
2674 independent reflections	*l* = −19→19

### Refinement

**Table:**

Refinement on *F*^2^	Primary atom site location: structure-invariant direct methods
Least-squares matrix: full	Secondary atom site location: difference Fourier map
*R*[*F*^2^ > 2σ(*F*^2^)] = 0.033	Hydrogen site location: inferred from neighbouring sites
*wR*(*F*^2^) = 0.092	H-atom parameters constrained
*S* = 1.05	*w* = 1/[σ^2^(*F*_o_^2^) + (0.0668*P*)^2^ + 0.103*P*] where *P* = (*F*_o_^2^ + 2*F*_c_^2^)/3
2674 reflections	(Δ/σ)_max_ = 0.001
258 parameters	Δρ_max_ = 0.30 e Å^−^^3^
1 restraint	Δρ_min_ = −0.18 e Å^−^^3^

### Fractional atomic coordinates and isotropic or equivalent isotropic displacement parameters (Å^2^)

**Table:**

	*x*	*y*	*z*	*U*_iso_*/*U*_eq_	
O1	0.10395 (12)	0.50018 (19)	0.12290 (8)	0.0237 (3)	
O2	0.24166 (13)	0.5963 (2)	0.03933 (8)	0.0286 (3)	
O3	0.25451 (12)	0.95997 (17)	0.77203 (8)	0.0214 (3)	
C1	0.49210 (17)	0.7214 (2)	0.92678 (10)	0.0213 (3)	
H1C	0.5064	0.8353	0.9597	0.026*	
H1D	0.4618	0.6239	0.9596	0.026*	
C2	0.58913 (18)	0.6782 (2)	0.87028 (11)	0.0206 (3)	
H2	0.6654	0.7638	0.8683	0.025*	
C3	0.6102 (2)	0.4859 (3)	0.85413 (12)	0.0307 (4)	
H3C	0.6701	0.4288	0.9112	0.037*	
H3D	0.6561	0.4701	0.8050	0.037*	
C4	0.4587 (2)	0.4096 (2)	0.82473 (12)	0.0308 (4)	
H4C	0.4323	0.3696	0.8793	0.037*	
H4D	0.4524	0.3084	0.7829	0.037*	
C5	0.35775 (17)	0.5589 (2)	0.77468 (10)	0.0193 (3)	
C6	0.2085 (2)	0.5381 (3)	0.78560 (12)	0.0312 (4)	
H6D	0.2140	0.5544	0.8504	0.047*	
H6E	0.1719	0.4203	0.7655	0.047*	
H6F	0.1443	0.6263	0.7479	0.047*	
C7	0.43341 (16)	0.7250 (2)	0.82255 (10)	0.0158 (3)	
C8	0.39518 (15)	0.9004 (2)	0.77576 (10)	0.0153 (3)	
H8	0.4669	0.9897	0.8092	0.018*	
C9	0.23898 (18)	0.9981 (3)	0.86009 (12)	0.0258 (4)	
H9D	0.1484	1.0596	0.8522	0.039*	
H9E	0.3183	1.0726	0.8949	0.039*	
H9F	0.2395	0.8882	0.8937	0.039*	
C10	0.39576 (16)	0.8908 (2)	0.67622 (10)	0.0158 (3)	
H10C	0.4951	0.8746	0.6749	0.019*	
H10D	0.3600	1.0032	0.6451	0.019*	
C11	0.30351 (15)	0.7405 (2)	0.62360 (10)	0.0133 (3)	
H11	0.2021	0.7609	0.6217	0.016*	
C12	0.35449 (16)	0.5638 (2)	0.67192 (10)	0.0163 (3)	
H12	0.4553	0.5475	0.6715	0.020*	
C13	0.2681 (2)	0.4112 (2)	0.61596 (12)	0.0254 (4)	
H13C	0.1674	0.4218	0.6155	0.030*	
H13D	0.3059	0.2990	0.6466	0.030*	
C14	0.27319 (19)	0.4064 (2)	0.51608 (11)	0.0221 (3)	
H14C	0.3725	0.3848	0.5159	0.027*	
H14D	0.2129	0.3087	0.4827	0.027*	
C15	0.21989 (15)	0.5799 (2)	0.46676 (10)	0.0149 (3)	
C16	0.05902 (15)	0.6053 (3)	0.45320 (11)	0.0224 (3)	
H16D	0.0426	0.6124	0.5135	0.034*	
H16E	0.0058	0.5056	0.4186	0.034*	
H16F	0.0264	0.7141	0.4189	0.034*	
C17	0.31116 (15)	0.7284 (2)	0.52507 (10)	0.0136 (3)	
H17	0.4127	0.6996	0.5304	0.016*	
C18	0.27348 (18)	0.8908 (2)	0.46325 (11)	0.0190 (3)	
H18E	0.3526	0.9771	0.4791	0.023*	
H18F	0.1857	0.9479	0.4685	0.023*	
C19	0.24985 (18)	0.8151 (2)	0.36523 (10)	0.0212 (3)	
H19C	0.3268	0.8547	0.3403	0.025*	
H19D	0.1571	0.8552	0.3233	0.025*	
C20	0.25155 (15)	0.6111 (2)	0.37410 (10)	0.0155 (3)	
H20	0.3516	0.5709	0.3823	0.019*	
C21	0.15462 (16)	0.5210 (2)	0.28707 (10)	0.0188 (3)	
H21	0.0546	0.5633	0.2767	0.023*	
C22	0.1550 (2)	0.3196 (3)	0.29402 (12)	0.0285 (4)	
H22A	0.1066	0.2695	0.2333	0.043*	
H22B	0.1050	0.2838	0.3379	0.043*	
H22C	0.2538	0.2772	0.3153	0.043*	
C23	0.20266 (16)	0.5758 (2)	0.20495 (10)	0.0202 (3)	
H23D	0.2026	0.7054	0.1999	0.024*	
H23E	0.3007	0.5328	0.2126	0.024*	
C24	0.13665 (17)	0.5221 (2)	0.04415 (11)	0.0211 (3)	
C25	0.0256 (2)	0.4403 (4)	−0.03584 (12)	0.0402 (5)	
H25D	−0.0046	0.5256	−0.0862	0.060*	
H25E	−0.0570	0.4052	−0.0165	0.060*	
H25F	0.0659	0.3367	−0.0570	0.060*	

### Atomic displacement parameters (Å^2^)

**Table:**

	*U*^11^	*U*^22^	*U*^33^	*U*^12^	*U*^13^	*U*^23^
O1	0.0205 (5)	0.0370 (7)	0.0136 (5)	−0.0064 (5)	0.0052 (4)	−0.0046 (5)
O2	0.0325 (6)	0.0348 (7)	0.0209 (6)	−0.0102 (6)	0.0118 (5)	−0.0031 (6)
O3	0.0187 (5)	0.0283 (6)	0.0156 (5)	0.0068 (5)	0.0027 (4)	−0.0055 (5)
C1	0.0284 (8)	0.0204 (8)	0.0125 (7)	0.0008 (7)	0.0024 (6)	0.0001 (6)
C2	0.0216 (8)	0.0210 (8)	0.0158 (7)	0.0040 (6)	0.0008 (6)	0.0002 (6)
C3	0.0431 (11)	0.0242 (9)	0.0185 (8)	0.0145 (8)	0.0001 (7)	0.0013 (7)
C4	0.0559 (12)	0.0158 (8)	0.0159 (8)	0.0000 (9)	0.0034 (7)	0.0025 (7)
C5	0.0296 (8)	0.0149 (7)	0.0123 (7)	−0.0046 (7)	0.0046 (5)	0.0008 (6)
C6	0.0381 (10)	0.0397 (11)	0.0187 (8)	−0.0207 (9)	0.0129 (7)	−0.0035 (8)
C7	0.0185 (7)	0.0160 (7)	0.0123 (7)	0.0002 (6)	0.0037 (5)	−0.0004 (6)
C8	0.0151 (6)	0.0144 (7)	0.0157 (7)	0.0010 (6)	0.0038 (5)	−0.0010 (6)
C9	0.0210 (7)	0.0358 (10)	0.0205 (8)	0.0039 (7)	0.0063 (6)	−0.0081 (7)
C10	0.0202 (7)	0.0124 (7)	0.0152 (7)	−0.0004 (6)	0.0059 (5)	0.0006 (6)
C11	0.0148 (6)	0.0127 (7)	0.0126 (6)	0.0004 (5)	0.0045 (5)	0.0000 (5)
C12	0.0230 (7)	0.0125 (7)	0.0123 (7)	−0.0016 (6)	0.0039 (5)	0.0008 (6)
C13	0.0446 (10)	0.0127 (7)	0.0162 (8)	−0.0077 (7)	0.0053 (7)	0.0005 (6)
C14	0.0337 (9)	0.0132 (7)	0.0169 (8)	−0.0010 (7)	0.0039 (6)	−0.0014 (6)
C15	0.0177 (7)	0.0150 (7)	0.0128 (6)	−0.0004 (6)	0.0061 (5)	−0.0010 (6)
C16	0.0165 (7)	0.0333 (9)	0.0187 (7)	−0.0039 (7)	0.0071 (5)	−0.0034 (7)
C17	0.0151 (6)	0.0130 (6)	0.0129 (7)	0.0003 (6)	0.0046 (5)	0.0003 (6)
C18	0.0274 (8)	0.0150 (7)	0.0144 (7)	−0.0007 (6)	0.0060 (6)	0.0012 (6)
C19	0.0307 (8)	0.0195 (8)	0.0131 (7)	−0.0020 (7)	0.0064 (6)	0.0019 (6)
C20	0.0154 (6)	0.0182 (7)	0.0132 (7)	−0.0009 (6)	0.0050 (5)	−0.0014 (6)
C21	0.0183 (7)	0.0250 (8)	0.0133 (7)	−0.0040 (6)	0.0049 (6)	−0.0035 (6)
C22	0.0410 (10)	0.0262 (9)	0.0179 (8)	−0.0116 (8)	0.0085 (7)	−0.0057 (7)
C23	0.0201 (7)	0.0265 (8)	0.0134 (7)	−0.0044 (7)	0.0039 (5)	−0.0033 (6)
C24	0.0239 (7)	0.0236 (8)	0.0167 (7)	−0.0010 (6)	0.0076 (6)	−0.0023 (6)
C25	0.0399 (10)	0.0626 (15)	0.0185 (9)	−0.0212 (11)	0.0092 (7)	−0.0084 (9)

### Geometric parameters (Å, °)

**Table:**

O1—C24	1.3425 (19)	C12—C13	1.536 (2)
O1—C23	1.4491 (18)	C12—H12	1.0000
O2—C24	1.198 (2)	C13—C14	1.538 (2)
O3—C9	1.4247 (19)	C13—H13C	0.9900
O3—C8	1.4388 (18)	C13—H13D	0.9900
C1—C2	1.501 (2)	C14—C15	1.532 (2)
C1—C7	1.516 (2)	C14—H14C	0.9900
C1—H1C	0.9900	C14—H14D	0.9900
C1—H1D	0.9900	C15—C16	1.5425 (19)
C2—C3	1.509 (2)	C15—C17	1.545 (2)
C2—C7	1.524 (2)	C15—C20	1.5525 (19)
C2—H2	1.0000	C16—H16D	0.9800
C3—C4	1.533 (3)	C16—H16E	0.9800
C3—H3C	0.9900	C16—H16F	0.9800
C3—H3D	0.9900	C17—C18	1.530 (2)
C4—C5	1.549 (2)	C17—H17	1.0000
C4—H4C	0.9900	C18—C19	1.551 (2)
C4—H4D	0.9900	C18—H18E	0.9900
C5—C6	1.532 (2)	C18—H18F	0.9900
C5—C7	1.535 (2)	C19—C20	1.559 (2)
C5—C12	1.5562 (19)	C19—H19C	0.9900
C6—H6D	0.9800	C19—H19D	0.9900
C6—H6E	0.9800	C20—C21	1.540 (2)
C6—H6F	0.9800	C20—H20	1.0000
C7—C8	1.507 (2)	C21—C23	1.523 (2)
C8—C10	1.520 (2)	C21—C22	1.536 (3)
C8—H8	1.0000	C21—H21	1.0000
C9—H9D	0.9800	C22—H22A	0.9800
C9—H9E	0.9800	C22—H22B	0.9800
C9—H9F	0.9800	C22—H22C	0.9800
C10—C11	1.527 (2)	C23—H23D	0.9900
C10—H10C	0.9900	C23—H23E	0.9900
C10—H10D	0.9900	C24—C25	1.503 (2)
C11—C17	1.5279 (19)	C25—H25D	0.9800
C11—C12	1.542 (2)	C25—H25E	0.9800
C11—H11	1.0000	C25—H25F	0.9800
			
C24—O1—C23	116.28 (12)	C12—C13—H13C	109.0
C9—O3—C8	113.69 (12)	C14—C13—H13C	109.0
C2—C1—C7	60.67 (10)	C12—C13—H13D	109.0
C2—C1—H1C	117.7	C14—C13—H13D	109.0
C7—C1—H1C	117.7	H13C—C13—H13D	107.8
C2—C1—H1D	117.7	C15—C14—C13	110.86 (13)
C7—C1—H1D	117.7	C15—C14—H14C	109.5
H1C—C1—H1D	114.8	C13—C14—H14C	109.5
C1—C2—C3	116.65 (16)	C15—C14—H14D	109.5
C1—C2—C7	60.16 (10)	C13—C14—H14D	109.5
C3—C2—C7	108.47 (15)	H14C—C14—H14D	108.1
C1—C2—H2	119.1	C14—C15—C16	110.98 (14)
C3—C2—H2	119.1	C14—C15—C17	107.23 (12)
C7—C2—H2	119.1	C16—C15—C17	112.21 (13)
C2—C3—C4	104.22 (16)	C14—C15—C20	116.41 (13)
C2—C3—H3C	110.9	C16—C15—C20	109.75 (12)
C4—C3—H3C	110.9	C17—C15—C20	99.78 (11)
C2—C3—H3D	110.9	C15—C16—H16D	109.5
C4—C3—H3D	110.9	C15—C16—H16E	109.5
H3C—C3—H3D	108.9	H16D—C16—H16E	109.5
C3—C4—C5	106.63 (14)	C15—C16—H16F	109.5
C3—C4—H4C	110.4	H16D—C16—H16F	109.5
C5—C4—H4C	110.4	H16E—C16—H16F	109.5
C3—C4—H4D	110.4	C11—C17—C18	118.76 (13)
C5—C4—H4D	110.4	C11—C17—C15	114.60 (12)
H4C—C4—H4D	108.6	C18—C17—C15	104.30 (11)
C6—C5—C7	112.04 (14)	C11—C17—H17	106.1
C6—C5—C4	111.19 (15)	C18—C17—H17	106.1
C7—C5—C4	103.00 (12)	C15—C17—H17	106.1
C6—C5—C12	112.47 (13)	C17—C18—C19	103.31 (13)
C7—C5—C12	108.54 (12)	C17—C18—H18E	111.1
C4—C5—C12	109.15 (14)	C19—C18—H18E	111.1
C5—C6—H6D	109.5	C17—C18—H18F	111.1
C5—C6—H6E	109.5	C19—C18—H18F	111.1
H6D—C6—H6E	109.5	H18E—C18—H18F	109.1
C5—C6—H6F	109.5	C18—C19—C20	106.99 (13)
H6D—C6—H6F	109.5	C18—C19—H19C	110.3
H6E—C6—H6F	109.5	C20—C19—H19C	110.3
C8—C7—C1	118.20 (14)	C18—C19—H19D	110.3
C8—C7—C2	120.24 (14)	C20—C19—H19D	110.3
C1—C7—C2	59.17 (10)	H19C—C19—H19D	108.6
C8—C7—C5	119.34 (12)	C21—C20—C15	118.22 (12)
C1—C7—C5	117.30 (14)	C21—C20—C19	112.44 (13)
C2—C7—C5	107.80 (13)	C15—C20—C19	103.36 (12)
O3—C8—C7	113.39 (13)	C21—C20—H20	107.4
O3—C8—C10	105.61 (12)	C15—C20—H20	107.4
C7—C8—C10	110.70 (13)	C19—C20—H20	107.4
O3—C8—H8	109.0	C23—C21—C22	109.58 (14)
C7—C8—H8	109.0	C23—C21—C20	108.70 (12)
C10—C8—H8	109.0	C22—C21—C20	113.40 (14)
O3—C9—H9D	109.5	C23—C21—H21	108.3
O3—C9—H9E	109.5	C22—C21—H21	108.3
H9D—C9—H9E	109.5	C20—C21—H21	108.3
O3—C9—H9F	109.5	C21—C22—H22A	109.5
H9D—C9—H9F	109.5	C21—C22—H22B	109.5
H9E—C9—H9F	109.5	H22A—C22—H22B	109.5
C8—C10—C11	112.28 (12)	C21—C22—H22C	109.5
C8—C10—H10C	109.1	H22A—C22—H22C	109.5
C11—C10—H10C	109.1	H22B—C22—H22C	109.5
C8—C10—H10D	109.1	O1—C23—C21	107.87 (12)
C11—C10—H10D	109.1	O1—C23—H23D	110.1
H10C—C10—H10D	107.9	C21—C23—H23D	110.1
C10—C11—C17	111.29 (12)	O1—C23—H23E	110.1
C10—C11—C12	110.33 (11)	C21—C23—H23E	110.1
C17—C11—C12	108.11 (12)	H23D—C23—H23E	108.4
C10—C11—H11	109.0	O2—C24—O1	123.80 (15)
C17—C11—H11	109.0	O2—C24—C25	124.99 (15)
C12—C11—H11	109.0	O1—C24—C25	111.21 (14)
C13—C12—C11	110.47 (12)	C24—C25—H25D	109.5
C13—C12—C5	112.33 (13)	C24—C25—H25E	109.5
C11—C12—C5	113.93 (13)	H25D—C25—H25E	109.5
C13—C12—H12	106.5	C24—C25—H25F	109.5
C11—C12—H12	106.5	H25D—C25—H25F	109.5
C5—C12—H12	106.5	H25E—C25—H25F	109.5
C12—C13—C14	112.90 (14)		
			
C7—C1—C2—C3	−96.93 (17)	C4—C5—C12—C13	73.88 (17)
C1—C2—C3—C4	47.2 (2)	C6—C5—C12—C11	76.57 (17)
C7—C2—C3—C4	−18.01 (19)	C7—C5—C12—C11	−47.97 (17)
C2—C3—C4—C5	30.41 (18)	C4—C5—C12—C11	−159.54 (13)
C3—C4—C5—C6	−150.84 (15)	C11—C12—C13—C14	55.86 (19)
C3—C4—C5—C7	−30.67 (17)	C5—C12—C13—C14	−175.72 (14)
C3—C4—C5—C12	84.52 (16)	C12—C13—C14—C15	−56.96 (19)
C2—C1—C7—C8	−110.21 (16)	C13—C14—C15—C16	−67.31 (17)
C2—C1—C7—C5	95.32 (16)	C13—C14—C15—C17	55.57 (16)
C1—C2—C7—C8	106.81 (16)	C13—C14—C15—C20	166.22 (13)
C3—C2—C7—C8	−142.48 (15)	C10—C11—C17—C18	−55.54 (16)
C3—C2—C7—C1	110.71 (16)	C12—C11—C17—C18	−176.85 (13)
C1—C2—C7—C5	−111.68 (14)	C10—C11—C17—C15	−179.67 (12)
C3—C2—C7—C5	−0.96 (18)	C12—C11—C17—C15	59.02 (15)
C6—C5—C7—C8	−79.26 (17)	C14—C15—C17—C11	−59.41 (16)
C4—C5—C7—C8	161.16 (15)	C16—C15—C17—C11	62.70 (17)
C12—C5—C7—C8	45.53 (18)	C20—C15—C17—C11	178.85 (12)
C6—C5—C7—C1	74.92 (18)	C14—C15—C17—C18	169.08 (12)
C4—C5—C7—C1	−44.66 (17)	C16—C15—C17—C18	−68.81 (15)
C12—C5—C7—C1	−160.29 (13)	C20—C15—C17—C18	47.34 (14)
C6—C5—C7—C2	138.82 (14)	C11—C17—C18—C19	−163.91 (12)
C4—C5—C7—C2	19.24 (16)	C15—C17—C18—C19	−34.87 (15)
C12—C5—C7—C2	−96.40 (14)	C17—C18—C19—C20	8.70 (17)
C9—O3—C8—C7	65.98 (17)	C14—C15—C20—C21	79.65 (17)
C9—O3—C8—C10	−172.64 (14)	C16—C15—C20—C21	−47.44 (19)
C1—C7—C8—O3	−84.03 (16)	C17—C15—C20—C21	−165.44 (13)
C2—C7—C8—O3	−152.90 (13)	C14—C15—C20—C19	−155.41 (14)
C5—C7—C8—O3	69.91 (17)	C16—C15—C20—C19	77.50 (16)
C1—C7—C8—C10	157.49 (13)	C17—C15—C20—C19	−40.50 (14)
C2—C7—C8—C10	88.62 (16)	C18—C19—C20—C21	148.82 (13)
C5—C7—C8—C10	−48.57 (18)	C18—C19—C20—C15	20.22 (16)
O3—C8—C10—C11	−70.54 (16)	C15—C20—C21—C23	177.98 (14)
C7—C8—C10—C11	52.56 (16)	C19—C20—C21—C23	57.63 (17)
C8—C10—C11—C17	−177.44 (12)	C15—C20—C21—C22	−59.89 (19)
C8—C10—C11—C12	−57.44 (16)	C19—C20—C21—C22	179.76 (15)
C10—C11—C12—C13	−176.47 (13)	C24—O1—C23—C21	−174.41 (14)
C17—C11—C12—C13	−54.57 (16)	C22—C21—C23—O1	59.70 (18)
C10—C11—C12—C5	55.99 (16)	C20—C21—C23—O1	−175.88 (13)
C17—C11—C12—C5	177.89 (12)	C23—O1—C24—O2	1.3 (3)
C6—C5—C12—C13	−50.0 (2)	C23—O1—C24—C25	−179.22 (17)
C7—C5—C12—C13	−174.54 (13)		
